# Satellite DNAs and the evolution of the multiple X_1_X_2_Y sex chromosomes in the wolf fish *Hoplias malabaricus* (Teleostei; Characiformes)

**DOI:** 10.1038/s41598-024-70920-7

**Published:** 2024-09-02

**Authors:** Gustavo Akira Toma, Alexandr Sember, Caio Augusto Gomes Goes, Rafael Kretschmer, Fabio Porto-Foresti, Luiz Antônio Carlos Bertollo, Thomas Liehr, Ricardo Utsunomia, Marcelo de Bello Cioffi

**Affiliations:** 1 Departamento de Genética e Evolução, Universidade Federal de São Carlos, São Carlos, SP 13565-905 Brazil; 2 Laboratory of Fish Genetics, Institute of Animal Physiology and Genetics, Czech Academy of Sciences, 277 21 Liběchov, Czech Republic; 3 Faculdade de Ciências, UNESP, Bauru, SP 17033-360 Brazil; 4 Departamento de Ecologia, Zoologia e Genética, Instituto de Biologia, Universidade Federal de Pelotas, Pelotas, RS 96010-610 Brazil; 5 Jena University Hospital, Institute of Human Genetics, Friedrich Schiller University, 07747 Jena, Germany

**Keywords:** Meiosis, Sex trivalent, FISH, Multiple sex chromosomes, Satellitome, Genetics, Cytogenetics, Evolutionary biology, Genomics

## Abstract

Multiple sex chromosomes usually arise from chromosomal rearrangements which involve ancestral sex chromosomes. There is a fundamental condition to be met for their long-term fixation: the meiosis must function, leading to the stability of the emerged system, mainly concerning the segregation of the sex multivalent. Here, we sought to analyze the degree of differentiation and meiotic pairing properties in the selected fish multiple sex chromosome system present in the wolf-fish *Hoplias malabaricus* (HMA). This species complex encompasses seven known karyotype forms (karyomorphs) where the karyomorph C (HMA-C) exhibits a nascent XY sex chromosomes from which the multiple X_1_X_2_Y system evolved in karyomorph HMA-D via a Y-autosome fusion. We combined genomic and cytogenetic approaches to analyze the satellite DNA (satDNA) content in the genome of HMA-D karyomorph and to investigate its potential contribution to X_1_X_2_Y sex chromosome differentiation. We revealed 56 satDNA monomers of which the majority was AT-rich and with repeat units longer than 100 bp. Seven out of 18 satDNA families chosen for chromosomal mapping by fluorescence in situ hybridization (FISH) formed detectable accumulation in at least one of the three sex chromosomes (X_1_, X_2_ and neo-Y). Nine satDNA monomers showed only two hybridization signals limited to HMA-D autosomes, and the two remaining ones provided no visible FISH signals. Out of seven satDNAs located on the HMA-D sex chromosomes, five mapped also to XY chromosomes of HMA-C. We showed that after the autosome-Y fusion event, the neo-Y chromosome has not substantially accumulated or eliminated satDNA sequences except for minor changes in the centromere-proximal region. Finally, based on the obtained FISHpatterns, we speculate on the possible contribution of satDNA to sex trivalent pairing and segregation.

## Introduction

Multiple sex chromosomes arise, in most cases, from chromosomal rearrangements between autosomes and existing sex chromosomes^1–3^. This way, a new genomic portion becomes sex-linked and may both restart the sex chromosome differentiation process^4,5^ and provide genetic material for sex chromosome turnover and/or hybrid incompatibility between the populations differing by their sex chromosome systems^6–9^. The analysis of multiple sex chromosomes may hence provide important insights into early phases of restarted sex chromosome differentiation, spreading over the newly added autosomal part, and shed light on the drivers of chromosome fusion^1,3,10–12^.

The most prevalent configuration of multiple sex chromosomes recorded thus far in nature is ♀X_1_X_1_X_2_X_2_/♂X_1_X_2_Y created by centric or tandem fusion between a Y chromosome and an autosome^1,2,13,14^. The system is clearly distinguishable both by a consistent difference between male and female diploid chromosome number (2n), and a remarkably large biarmed (meta- or submetacentric) neo-Y chromosome present in one copy in males and entirely missing in females. While neo-Y hence achieves instant heteromorphy (i.e. it can be distinguished cytologically), the process is not analogous to heteromorphy achieved by a gradual sequence differentiation and gene content degradation^15^. Hence, in multiple sex chromosome systems, the heteromorphy does not necessarily correlate with the degree of sex chromosome differentiation^3^. To date, out of the 81 known cases of fish multiple sex chromosomes (^2,16–19^ and references therein), 65 are of the X_1_X_2_Y type and the overwhelming majority of them exhibits signs of low differentiation, inferred mostly from the degree of repetitive DNA and heterochromatin accumulation^2,17,18^.

Y-autosome fusion results in the formation of a sex trivalent in the male first meiotic division. Its correct pairing and segregation are critical for stability and fixation as a multiple sex chromosome system given that deviations from these processes may lead to meiotic arrest or reduced fertility and/or viability of the progeny^3,5,20,21^. Although the properties of sex trivalents have been investigated in diverse eukaryotic lineages^22–27^ (including fishes^28,29^), little is known about the mechanisms related to their correct alignment and segregation, and the role of repetitive DNAs in these processes.

Repetitive DNA, i.e. the highly variable fraction of the genome present in multiple dispersed or tandemly arrayed copies, has a multilayered influence on the evolution of sex chromosomes^30–33^. Firstly, repetitive DNAs usually populate the region of abolished recombination on sex chromosomes, thus contributing to gene content decay and further spread of the region of differentiation on the Y (or, by analogy, W) chromosome by repeat amplification and by triggering heterochromatinization^30–34^. Secondly, repetitive DNA may induce intrachromosomal rearrangements via non-allelic homologous recombination which may lead, e.g., to inversion or deletion and this way to a further spread of non-recombining region and/or—similarly to repeat amplification—potentially to Y/W sex chromosome heteromorphy^4,35^. Thirdly, repeat content may also promote sex chromosome-autosome fusions^36,37^ or shield the newly added autosomal part from the effect of dosage compensation if given enough time^23,38,39^. Last but not least, certain repeats have been found to directly rewire the expression patterns within the sex determination regulatory network^40,41^ and to facilitate sex chromosome pairing in meiosis^21,42^.

Satellite DNA (satDNA) is a highly abundant repetitive DNA class with an extreme diversity in monomer size and nucleotide sequence as well as distribution patterns (mostly tandemly organized but also dispersed, present both in heterochromatin and euchromatin)^43–45^. As these repeats often occupy key chromosomal regions such as centromeres and telomeres, satDNA sequences play important roles in chromosome architecture and function, including centromere organization and kinetochore assembly, which allow proper chromatid cohesion and segregation in cell division^46–48^. SatDNAs are among the fastest evolving sequences in the genome and therefore they generate highly species-specific landscapes in terms of quantity, distribution, and diversity of satDNA monomer collection (the so-called satellitome)^43,44,49,50^. During the past decade, the advancements in bioinformatic tools allowed a steep increase in studies aimed at satellitome characterization and its subsequent mapping on chromosomes by fluorescence in situ hybridization (FISH)^43,49,51^. Given the above-mentioned satDNA properties, this type of investigation has already provided important novel insights into satDNA evolution at inter-specific and inter-population levels^52–55^, chromosomal markers for genomic composition of hybrids and polyploids^56,57^ (peri)centromeric repeat turnover^58–60^ and the evolutionary dynamics of sex^61,62^ and B chromosomes^63^, with all of these aspects already being studied also in teleost fishes^64–70^.

In the present work, we aimed to analyze the differentiation and meiotic pairing properties of a selected X_1_X_2_Y multiple sex chromosome system through the lens of satellitome analysis. For this, we chose the wolf-fish *Hoplias malabaricus* (Characiformes, Erythrinidae) as a model. This species complex (hereafter abbreviated as HMA) comprises seven karyotype forms (karyomorphs) denoted A–G, which represent populations differing by their karyotype features, including the presence of four different karyomorph-specific sex chromosome systems (based on their configuration and degree of differentiation): (1) a well-differentiated, heteromorphic XX/XY (karyomorph HMA-B), non-homologous to any of the following sex chromosome systems), (2) nascent XX/XY systems (HMA-C and HMA-F; these are mutually non-homeologous), and two multiple sex chromosome systems: (3) X_1_X_1_X_2_X_2_/X_1_X_2_Y in HMA-D (derived from XX/XY of HMA-C), and (4) XX/XY_1_Y_2_ in HMA-G) (derived from XX/XY of HMA-F)^71–74^.

Molecular cytogenetic analyses showed that the X and Y chromosomes of HMA-C differ just slightly by degree of their (peri)centromeric repeats amplification, and that the X_1_X_1_Y system of HMA-D evolved from HMA-C XY system through a tandem (end-to-end) fusion between the short (p) arms of a Y chromosome and a p-arms of one homolog from autosome pair 20^74^. Meiotic analysis indicated that the neo-Y, the X_1_ and X_2_ chromosomes pair regularly by forming a trivalent without obvious irregularities in the first meiotic division^28^. This was further corroborated by repetitive DNA analysis^74^, whole chromosome painting (WCP)^72^ and comparative genomic hybridization (CGH)^75^. However, a detailed study on the sequence differentiation on these multiple sex chromosomes is still missing.

We herein analyzed the satellitome of HMA-D karyomorph by a combination of genomic and cytogenetic approaches. We focused specifically on the satDNA landscape on X_1_X_2_Y multiple sex chromosomes to trace the origin and pathways of differentiation of this system. To determine whether there was a significant change in the Y-linked satDNA patterns after the Y-autosome fusion, we also investigated the chromosomal distribution of selected satDNA monomers in the homeologous XX/XY sex chromosome system of HMA-C. The results showed that the neo-Y chromosome has not yet undergone major changes in its satDNA landscape after the fusion event which corroborates the previous assumptions that this multiple sex chromosomes is still poorly differentiated.

## Results

### Satellitome features of HMA-D

Based on the five subsequent rounds of analysis via Tandem Repeat Analyzer (TAREAN) pipeline^51^ we revealed 56 satDNA monomers in HMA-D (Supplementary Table 1). Among them, HmaSat03 was the only one characterized previously^76^, corresponding to the 5S*Hin*dIII-DNA sequence. Forty HmaSatDNA monomers had the proportion of AT-pairs over 50%. The repeat unit lengths (RUL) varied from 6 to 2774 bp (average of 493 bp), with 39 HmaSatDNA monomers being shorter, and 17 ones longer than 100 bp (Supplementary Table 1). By calculating the sex abundance ratio (M/F) (see Material and Methods section for explanation) we found seven male (HmaSat07; HmaSat14; HmaSat18; HmaSat32; HmaSat34; HmaSat42; HmaSat56) and six female-biased HmaSatDNAs (HmaSat11; HmaSat17; HmaSat25; HmaSat36; HmaSat48; HmaSat51) (Supplementary Table 1). The homology search between HmaSatDNAs revealed the presence of five superfamilies (SF1-SF5) (Supplementary Table 1).

### Hybridization patterns of selected HmaSatDNA monomers in the HMA-D karyomorph

**Fig. 1:**
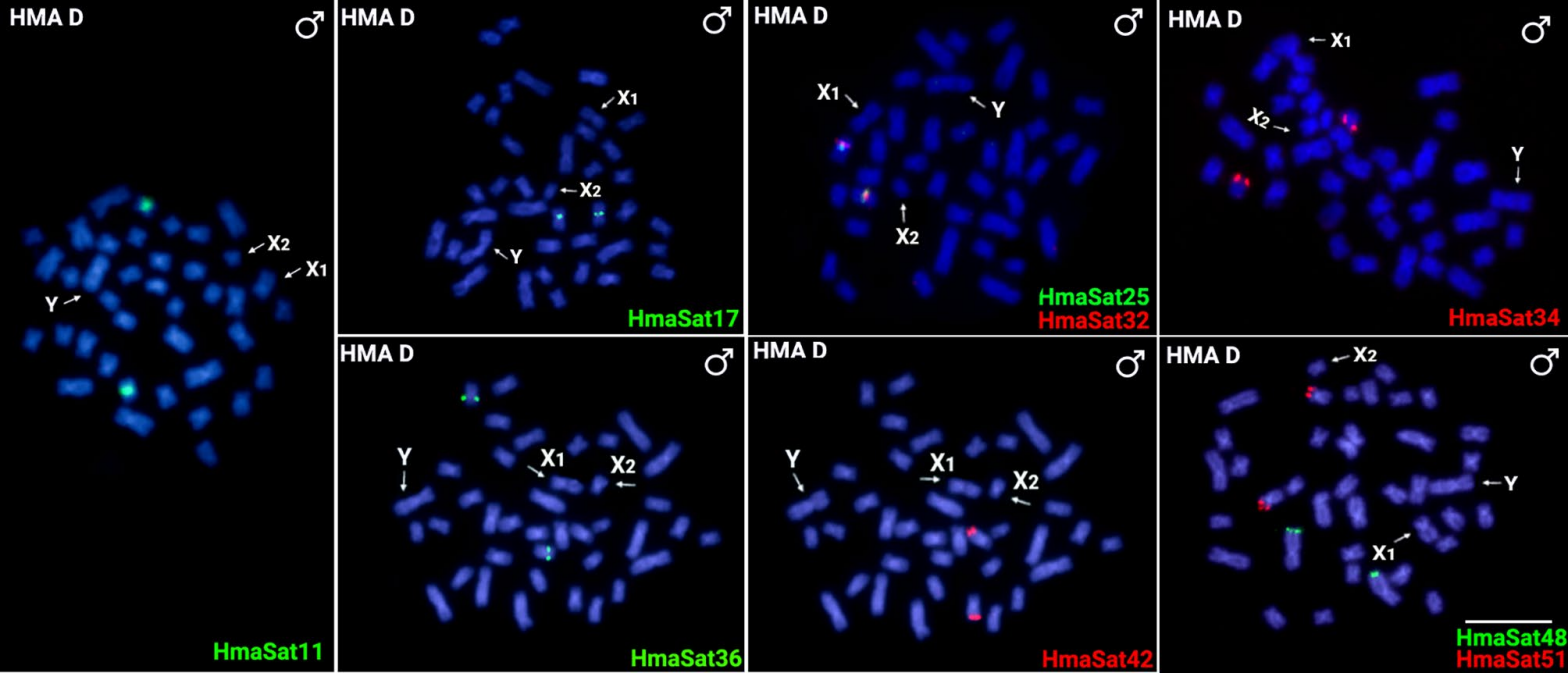
Male mitotic metaphases of *Hoplias malabaricus*, karyomorph D (HMA-D) after FISH with a set of HmaSatDNA monomers located exclusively on autosomes. SatDNA family names are indicated on the right bottom, in green (Atto-488-dUTP) or red (Atto-550-dUTP). The arrows indicate the X_1_, X_2,_ and neo-Y sex chromosomes. Bar = 5 μm.

**Fig. 2:**
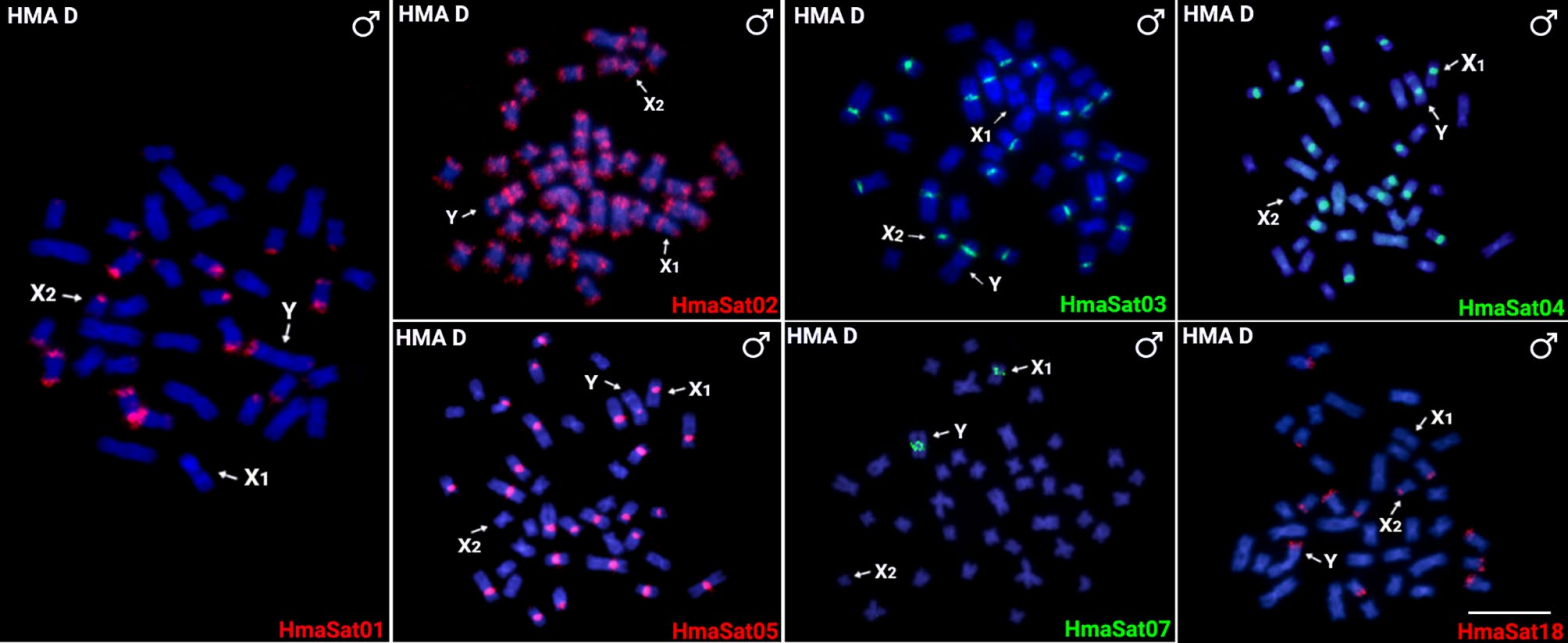
Male mitotic metaphases of *Hoplias malabaricus*, karyomorph D (HMA-D) after FISH with a set of HmaSatDNA monomers located both on autosomes and sex chromosomes. SatDNA family names are indicated on the right bottom, in green (Atto-488-dUTP) or red (Atto-550-dUTP). The arrows indicate the X_1_, X_2_ and neo-Y sex chromosomes. Bar = 5 μm.

Among the 18 mapped HmaSatDNA monomers (marked by asterisk in Supplementary Table 1), only two (HmaSat14 and HmaSat56) did not produce specific signals on male and female mitotic chromosomes after FISH (data not shown). This outcome may be attributed to their potentially low abundance in the genome or their arrangement into clusters that are too small for effective cytological detection. The remaining examined satDNA monomers provided varied hybridization patterns (Figs. 1 and 2 and Supplementary Figs. 1 and 2). Only two hybridization signals, restricted to a certain autosome pair, were recorded for nine HmaSatDNA monomers, namely HmaSat11, HmaSat17, HmaSat25, HmaSat32, HmaSat34, HmaSat36, HmaSat42, HmaSat48, and HmaSat51, with HmaSat25 and HmaSat32 showing adjacent co-localized arrangement (Fig. 1 and Supplementary Figs. 1 and 2). On the other hand, FISH with HmaSat07 probe yielded strong (peri)centromeric signals exclusively on sex chromosomes, extended towards the p- and q-arms of the X_1_ and neo-Y, respectively (Fig. 2 and Supplementary Figs. 1 and 2). HmaSat07 was hence the only sex chromosome-specific satDNA monomer revealed in the present study. Finally, the remaining six satDNA monomers displayed FISH signals on multiple autosomes, neo-Y, and either X_1_ or X_2_ sex chromosomes. HmaSat01 and HmaSat18 exhibited highly similar patterns, with strong clusters being detected in the telomeric regions of many autosomes along with X_2_ and neo-Y chromosomes. On the other hand, HmaSat03, HmaSat04 and HmaSat05 shared the pattern of (peri)centromeric signals, being present on many (HmaSat04) or almost all (HmaSat03 and HmaSat05) chromosomes of the complement, including neo-Y and either X_2_ (HmaSat03) or X_1_ (HmaSat04 and HmaSat05) chromosome. A specific pattern was produced by the probe for HmaSat02, with prominent clusters at many terminal and interstitial regions across the entire chromosome set (Fig. 2).

### Patterns of HmaSatDNA monomers on the meiotic sex trivalent formed by X_1_X_2_Y multiple sex chromosomes of HMA-D

**Fig. 3:**
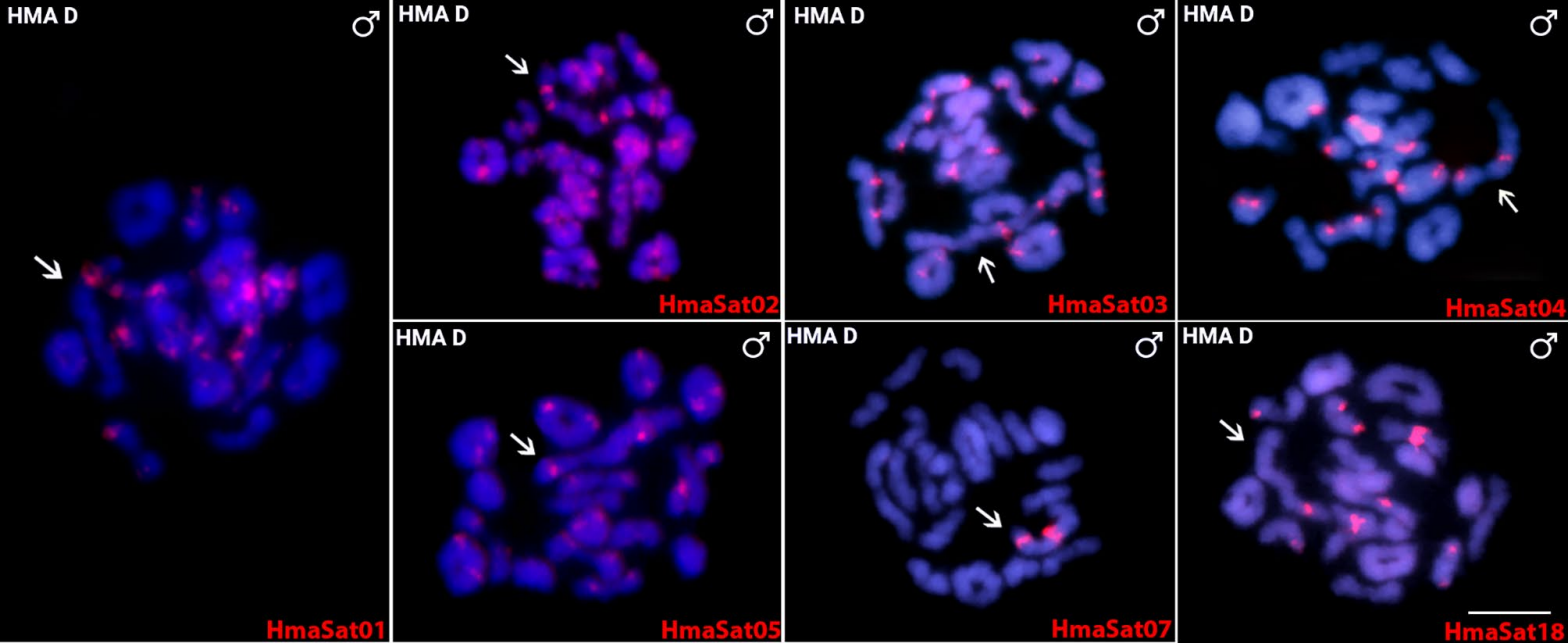
Meiotic chromosomes of male *Hoplias malabaricus* karyomorph D (HMA-D) after FISH with a set of HmaSatDNA monomers located both on autosomes and sex chromosomes. Arrows indicate the sex trivalent. Bar = 5 μm.

To better understand the organization of HmaSatDNA monomers on the X_1_X_2_Y multiple sex chromosomes of HMA-D during their meiotic pairing into the trivalent structure, we sought to map the seven HmaSatDNAs that display detectable clusters on these sex chromosomes (Fig. 2). A prominent cluster was found for the HmaSat01 and HmaSat18 monomers, corresponding to the segment formed by the terminal regions of the X_2_ and neo-Y chromosomes (Fig. 3). Four sites that resided on the X_1_, X_2_, and neo-Y chromosomes were displayed by the HmaSat02-specific probe. In contrast, the HmaSat03 probe displayed two signals, one of which populated the q-arms of the neo-Y chromosome and the second located in the centromeric region of the X_2_ chromosome (Fig. 3). All the three remaining HmaSatDNA monomers (HmaSat04, HmaSat05, and HmaSat07) presented two FISH signals in the (peri)centromeric region of the X_1_ and neo-Y chromosomes (Fig. 3).

### Chromosomal location of HmaSatDNA monomers in HMA-C

**Fig. 4:**
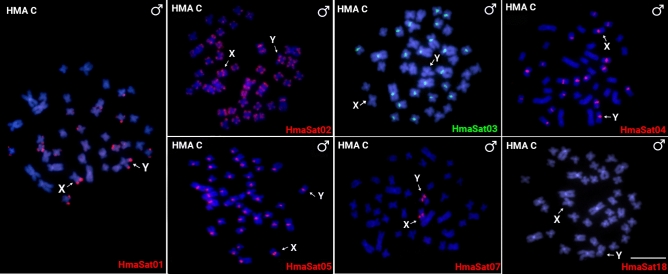
Male mitotic metaphases of *Hoplias malabaricus*, karyomorph C (HMA-C) after FISH with a set of HmaSatDNA monomers that produced detectable signals on X_1_X_2_Y sex chromosomes of karyomorph D (HMA-D). SatDNA family names are indicated on the right bottom, in green (Atto-488-dUTP) or red (Atto-550-dUTP). The arrows point on the X and Y chromosomes. Bar = 5 μm.

We mapped the subset of seven HmaSatDNA monomers, which hybridized to HMA-D sex chromosomes, also to metaphases of *H. malabaricus* males of karyomorph C (HMA-C) to examine their possible presence on nascent XY sex chromosomes (Fig. 4 and Supplementary Fig. 3). Most of FISH signals resembled the patterns revealed on the HMA-D sex chromosomes, with the exception of HmaSat01 (Fig. 2). The same satDNA monomers found on the X_1_ and the (peri)centromeric region of the neo-Y, (HmaSat02; HmaSat04; HmaSat05; HmaSat07) were located in the (peri)centromeric regions of the corresponding X and Y chromosomes in the karyomorph HMA-C (Fig. 4 and Supplementary Fig. 3). In a similar way, the HmaSat01 and HmaSat02 monomers, with signals on the X_2_ and neo-Y (HmaSat01), or all multiple sex chromosome (HmaSat02) of the HMA-D, also formed telomeric signals in both nascent sex chromosomes of the HMA-C (Fig. 4 and Supplementary Fig. 3). In turn, the HmaSat03 and HmaSat18 monomers were the only ones without detectable hybridization signals on the X and Y chromosomes of HMA-C. In the case of HmaSat03, the situation particularly reflects the fact that clusters of this satDNA reside on the X_2_ sex chromosome of HMA-D, i.e. the newly added chromosomal part (Fig. 2), therefore this satDNA joined the sex chromosome system only after the Y-autosome fusion. On the other hand, HmaSat18 was entirely absent from the HMA-C chromosomes, at least under the FISH resolution (Fig. 4 and Supplementary Fig. 3). Lastly, it is worth noting, that the probe corresponding to HmaSat05 monomer marked the centromeric regions of all but one chromosome pair in the HMA-C complement, which, after comparison with the HMA-D pattern (particularly the lack of signal on X_2_), indicates that the sole chromosome pair without the signal represents highly probably the linkage group (pair 20) which has been incorporated into the X_1_X_2_Y system in karyomorph HMA-D.

### Minimum spanning trees (MST)

**Fig. 5:**
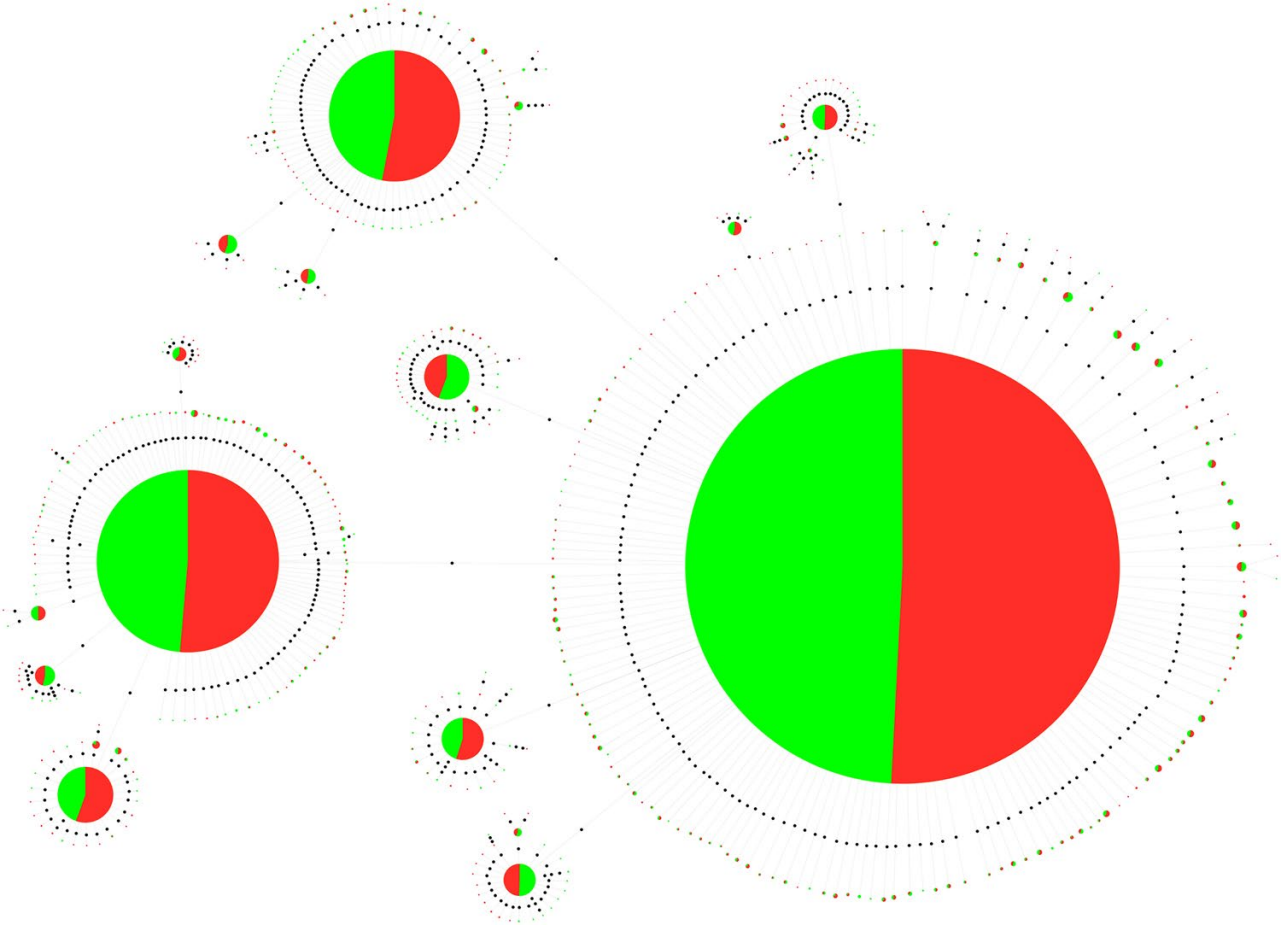
Linear MST of HmaSat02 satDNA obtained from female (red) and male (green) reads. The diameter of the circle is proportional to the abundance of the haplotype. Black circles represent one base of divergence between haplotypes.

**Fig. 6:**
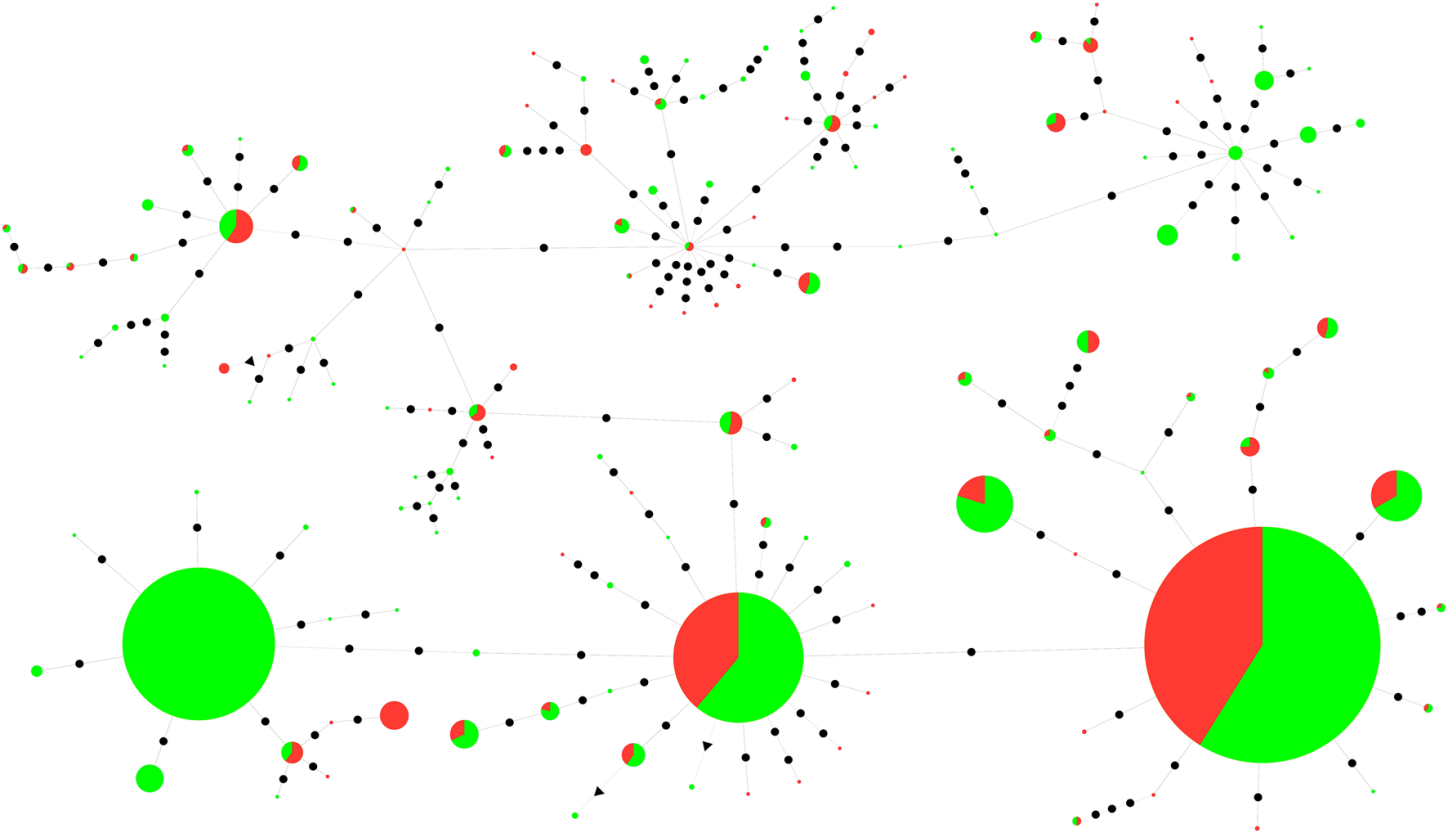
Linear MST of HmaSat18 satDNA obtained from female (red) and male (green) reads. The diameter of the circle is proportional to the abundance of the haplotype. Black circles represent one base of divergence between haplotypes.

We selected two satDNA monomers to generate minimum spanning trees: (1) the HmaSat02, as the satDNA with the most pronounced hybridization pattern, with telomeric and interstitial signals located on all chromosomes of the HMA-D complement, including multiple sex chromosome system, and (2) the HmaSat18, which displayed terminal signals on several autosomes and in the X_2_ and neo-Y chromosomes of this karyomorph. The MST of HmaSat02 revealed the predominance of three abundant haplotypes, besides the presence of many monomers with few mutations. All of them displayed a very similar abundance to all haplotypes identified in males and females (Fig. 5). The MST of HmaSat18 revealed the presence of many haplotypes. Although the predominant ones were shared between males and females, unique male-specific haplotypes were observed (Fig. 6).

## Discussion

In this work, we studied the satDNA content and its chromosomal distribution in one cytotype (karyomorph D) of the wolf-fish *H. malabaricus* with multiple sex chromosome system of the X_1_X_2_Y type. The main purposes of this study were to investigate, (1) whether certain satDNAs could be associated with the breakpoint regions involved in the Y-autosome fusion which gave rise to the mentioned multiple sex chromosome system, and (2) whether the neo-Y chromosome already underwent a significant degree of differentiation that could be reflected by the satDNA accumulation. The opportunity to compare the satDNA distribution also with the patterns on the XY sex chromosomes of closely related karyomorph (HMA-C), from which X_1_X_2_Y of HMA-D originated via Y-autosome fusion^74^, qualifies *H. malabaricus* as a useful study model for these research questions.

A recent burst in satellitome studies has led to significant advancements in our understanding of the teleost satDNA evolutionary dynamics and its possible impact on various genomic processes^66,68–70,77^. Thus far, a particular attention has been given to characiform fishes^64,66–68,77^. Among the characiform satellitomes, the diversity of satDNA ranges from 30 (*Piaractus mesopotamicus*) to an exceptional diversity of 164 monomers (*Megaleporinus macrocephalus*)^68,78^. In the present study, we revealed 56 satDNA monomers in the genome of *H. malabaricus*—karyomorph D (Supplementary Table 1), which falls within the above-mentioned range reported for characiforms. We further revealed an extraordinary wide range of satDNA monomer sizes, ranging from 6 to 2774 bp, with the predominance of those exhibiting higher proportion of AT-pairs (> 50%) (Supplementary Table 1), similarly to what has been found in some other vertebrates such as mammals and amphibians^79–81^, but differing from reptiles and birds with a predominantly CG-rich content^82,83^.

**Fig. 7:**
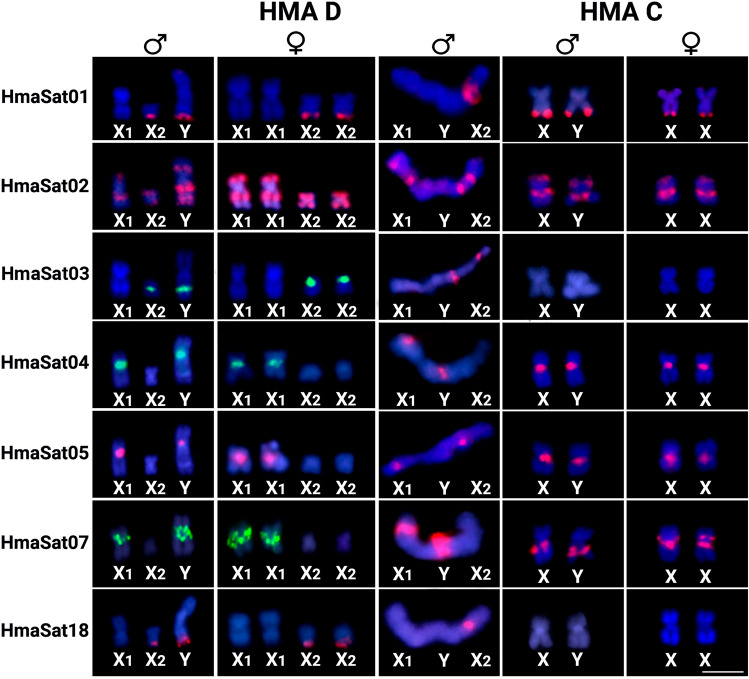
Partial karyotypes of sex chromosomes from *H. malabaricus* karyomorphs HMA-D and HMA-C after FISH with selected satDNA monomers, including the patterns on X_1_X_2_Y sex chromosome meiotic trivalent (HMA-D). The sex chromosomes were retrieved from chromosome spreads presented in Figs. 2, 3 and 4. Bar = 5 μm.

Sixteen out of 18 chromosomally mapped HmaSatDNA monomers showed detectable clusters pointing on their organization into high-copy number arrays. We observed the following three major distribution patterns: (1) only two hybridization signals restricted to a certain autosome pair (nine monomers; Fig. 1); (2) clusters located on the X_1_, X_2_, and/or neo-Y sex chromosomes in addition to several-to-all autosomes (six monomers; Figs. 2, 7), and (3) a single satDNA, HmaSat07, accumulated exclusively in the pericentromeric regions and surrounding area on the X_1_ and neo-Y sex chromosome (Figs. 2, 7).

Intriguingly, although the most abundant satDNA sequence in the genome very often occupies centromeric regions of all chromosomes, implying its functional significance for this crucial chromosomal region^84^, our data on distribution patterns imply this is not the case for HmaSat01, the most abundant satDNA monomer in HMA-D. Previously, the 5S*Hin*dIII-DNA satDNA, corresponding to the herein characterized HmaSat03 monomer, has been proposed to play a role in centromere function or structure in this fish^76^, however, our current analysis showed that other satDNA, namely HmaSat05, is a more likely candidate for this role based on the higher proportion of (peri)centromeric signals per chromosome set (Fig. 2). Yet, this monomer does not form detectable clusters on each centromeric region in the complement, with a single autosome pair (putatively the autosomal addition creating X_1_X_2_Y system in HMA-D) lacking the signal in HMA-C, and more chromosomes without the signal in HMA-D (Figs. 1, 2, 4). Our present findings therefore suggest a gradual change in the (peri)centromeric satDNA content in *H. malabaricus*, with analogous pattern being recently reported for African *Nothobranchius* killifishes^85^. In *H. malabaricus*, this process seems to be considerably fast given that HmaSat05 monomer occupies the centromeric regions only in HMA-C and HMA-D, while it is not present in these regions on chromosomes of closely related karyomorphs A, F, and G (Supplementary Fig. 4). It is generally thought that the mechanisms driving fast turnover of centromeric repeats and associated proteins may be responsible for a reproductive barrier between diverging populations of the same or closely related species, due to hybrid incompatibility related to chromosome segregation^86–88^. This, together with possible incompatibilities related to different sex chromosome systems^89^, might have contributed to the previously reported absence of hybrids between *H. malabaricus* karyomorphs^90^, thus supporting the proposal that the karyomorphs of *H. malabaricus* represent already distinct evolutionary units^71^. A functional role of HmaSat05 monomer in HMA-C and HMA-D centromeres will need to be further assessed by testing the interaction between this satDNA and key centromeric proteins such as CENP-A^52,58^.

From this point on, we will attempt to address the two main queries raised beforehand, starting with the inquiry of whether changes in satDNAs content accompanied the rearrangement(s) involved in the origin of the X_1_X_2_Y multiple sex chromosome system present in HMA-D.

The association between repetitive DNA accumulation and the occurrence of chromosomal rearrangements along with overall elevated genome instability has been extensively recorded in diverse lineages across the tree of life^91,92^, including humans^93^. In the case of X_1_X_2_Y sex chromosome system of HMA-D, most neo-Y-linked clusters of HmaSatDNA monomers are concentrated in the centromeric region and its surrounding area (Fig. 7). From the herein mapped monomers, only HmaSat02 and HmaSat03 repeats populate the putative breakpoint junction of the Y-autosome tandem fusion (Figs. 2 and 3), which has been inferred from previous WCP experiments^72^, and comparisons of the position of repetitive DNA clusters between the X_2_ and neo-Y (HMA-D) and the ancestral XY sex chromosomes in HMA-C^74^. Whether or not these sequences directly contributed to the rearrangement remains an open question. The other monomers, HmaSat04 and HmaSat05, forming strong (peri)centromeric accumulations on X_1_ and neo-Y, are likely absent from the ancestor of the X_2_ chromosome as demonstrated by the lack of FISH signals in Figs. 2 and 3. Contrary to other studies^61,62^, which evidenced a high rate of elimination and turnover of satDNAs populating neo-sex chromosomes, our findings indicate that both HmaSat04 and HmaSat05 satDNAs were not excised during the Y-autosome fusion event or lost through the subsequent process of centromere inactivation/elimination (reviewed by Barra and Fachinetti^94^, and McKinley and Cheeseman^95)^.

The second question was whether or not the neo-Y chromosome underwent a significant degree of differentiation, which could be inferred from the level of satDNA accumulation. For this, we took the opportunity of direct comparison of satDNA landscapes between the multiple X_11_X_2_Y sex chromosomes of HMA-D and standard XY sex chromosome system of HMA-C from which the system of HMA-D evolved. Species with heteromorphic sex chromosomes usually exhibit sex-linked variations in the accumulation of certain repetitive DNA sequences; they can be either substantially enriched or even limited to sex chromosome(s)^31,32,43,61,62^, which has been reported also in teleosts^64,67,68^. When narrowed down to multiple sex chromosomes, those so far revealed in teleost fishes show, in the overwhelming majority, low degree of repeat accumulation^2^. In the case of X_1_X_2_Y system of HMA-D, our results corroborate the previous findings from other FISH-based studies^73–75^ by showing non-elevated degree of satDNA accumulation, with a limited difference between the synteny blocks of X_1_/X_2_ versus neo-Y chromosome (Supplementary Table 1; Fig. 7). Minor changes linked to neo-Y satDNA content may be related to the loss of the HmaSat01 accumulation from the terminal portion of q-arms (on both neo-Y and X_1_ chromosome), based on the comparison with patterns found in the XY sex chromosome system of HMA-C (see Fig. 7). Nevertheless, it is not possible to rule out the alternative hypothesis that HmaSat01 repeats populated/amplified to cytologically detectable extent on the XY sex chromosomes of HMA-C more recently, i.e. the sex chromosome-autosome fusion in HMA-D might predate this accumulation.

The causal relationship between repetitive DNA accumulation and recombination suppression on sex chromosomes has been a matter of a long-standing debate. It seems more likely that repetitive DNA accumulates in the region of suppressed recombination and then it contributes to further expansion of this region, to genetic decay, and to sex chromosome heteromorphy^4,30–35,96^. Important question is then: what happens during the first meiotic division when sex chromosomes need to pair efficiently despite possible differences in size, morphology and/or repetitive DNA/heterochromatin content? Accumulation of repeated DNAs and heterochromatin could hamper the establishment and stability of the trivalent, preventing thereby its proper segregation, leading to high frequency of unbalanced gametes and thus being avoided by natural selection^20–22,28,97,98^. Bearing this in mind, we speculate that the lack of major differences in satDNA accumulation between the members of X_1_X_2_Y sex chromosome system of HMA-D, along with generally low degree of repeat accumulation might have contributed to faithful pairing and segregation of these sex chromosomes (Fig. 7). The presence of sex chromosome-specific repeat segments in the area of assumed sequence differentiation, both in the XY sex chromosome system of HMA-C and X_1_X_2_Y system of HMA-D, might facilitate smooth sex chromosome pairing within this region, without asynapsis, as has been already proposed for other studied teleost fishes^99,100^_._

## Conclusions

Taken together, our present study provides novel insight into the aspects linked to emergence and differentiation of representative fish multiple sex chromosome system—X_1_X_2_Y present in *H. malabaricus*, karyomorph D. We showed that the satDNA content remained rather unchanged during the evolution of this system after Y-autosome fusion. Last but not least, by unravelling the satellitome features of this *H. malabaricus* karyomorph, we provided new tools for further studies on satDNA evolution in characiform fishes.

## Material and methods

### Sampling and chromosome preparation

We analyzed 20 individuals of *H. malabaricus* karyomorph HMA-C (11 males; 9 females) and 17 individuals of karyomorph HMA-D (10 males; 7 females). HMA-C individuals were collected in Bento Gomes River (Poconé, Mato Grosso State, Brazil) and HMA-D in the Monjolinho stream (São Carlos, São Paulo State, Brazil). The sex of sampled individuals was identified by morphological analysis of gonadal tissues, and each individual was assigned to specific karyomorph based on the characteristic cytogenetic profiles (e.g., 2n, karyotype structure) uncovered in previous studies^71,73,74^. Sampling was authorized by the Brazilian Environmental Agency ICMBIO/SISBIO (License 48628-14) and SISGEN (A96FF09). Mitotic chromosomes were obtained from kidney biopsies following the protocol by Bertollo et al.^101^, while meiotic spreads were prepared from testes of HMA-D males according to Kligerman and Bloom^102^. Briefly, we treated the animals with benzocaine and applied a colchicine solution (0.025%) for 40 min. Subsequently, we excised kidney samples and testicular tissue, which were fragmented and treated with a hypotonic solution (0.075 M KCl) for 20 min at 37 °C. Both mitotic cell suspensions and fragmented testes were preserved in Carnoy I fixative (3:1 methanol/glacial acetic acid). Chromosome slides were stained with a 10% Giemsa solution (pH 6.8). All experiments followed the ethical guidelines sanctioned by the Ethics Committee on Animal Experimentation of the Universidade Federal de São Carlos (Process Number 7994170423). The authors complied with ARRIVE guidelines.

### DNA extraction and next generation sequencing

Genomic DNA (gDNA) of one male and one female of HMA-D was extracted using liver tissue by the standard phenol–chloroform-isoamylalcohol procedure^103^. Illumina paired-end libraries with 150 bp read length were prepared from the isolated HMA-D gDNAs and sequenced on the BGISEQ-500 platform at BGI (BGI Shenzhen Corporation, Shenzhen, China). The sequencing yielded a total of 1.55 Gb and 1.4 Gb of raw reads for males and females, respectively. The genomic reads obtained were deposited in the Sequence Read Archive (SRA) under accession numbers SRR27786732 (male) and SRR27786731 (female).

### Analysis of *H. malabaricus* satDNAs

We characterized the satellite DNA content of HMA-D male and female independently. First, we examined the read quality (Q > 20 for all nucleotides), by using the Trimmomatic software version 3.0^104^. Then, a subset of 2 × 500,000 reads has been randomly subsampled and usedas input for the TAREAN analysis^51^. All recovered satDNAs were removed from the original library by using the DeconSeq software version 0.4.3^105^, and from the remaining dataset, a new randomly selected subset of 2 × 500,000 reads was created to pass through a second round of the TAREAN analysis. These steps (iterations) were repeated until no novel satDNAs were found. In the next step, we performed a screening and the removal of the non-satDNA repetitive DNA classes (e.g., multigene families, transposable elements etc.) from our dataset. Finally, we performed a homology search with RM_homology (https://github.com/fjruizruano/satminer/blob/master/rm_homology.py) to group the sequences into variants (> 95% similar), families (80–95% similar), and superfamilies (50–80% similar)^49^. The catalog of satellite DNAs was deposited on the GenBank with accession numbers PP262651-PP262706.

### Estimating the abundance and genetic distances of satDNAs

The abundance of each satDNA (proportion of reads containing the same repeat vs. the entire pool of reads) was estimated by selecting 2 × 500,000 reads from each genomic library (HMA-D female and HMA-D male). Then, using the “cross-match” tool in the RepeatMasker version 4.1.6 106 and a custom python script (https://github.com/fjruizruano/ngsprotocols/blob/master/repeat_masker_run_big.py, accessed on 20/01/2023), the reads were aligned against each respective catalog. We used the script calcDivergenceFromAlign.py in the RepeatMasker^106^ to calculate the genetic distance of each satDNA (Kimura-2-parameter). All satDNAs were named “HmaSat” and numbered according to their decreasing abundance in the genome. Finally, to check for the possible presence of male-biased satDNAs, we calculated the male/female (M/F) abundance ratio of each HmaSat monomer given by RepeatMasker and selected those with an M/F ratio greater than 1.0.

### Minimum spanning trees (MST)

To achieve the accurate scores of monomer diversity, we used PHYLOVIZ version 2.0^107^ to construct the minimum spanning trees (MSTs) for two satDNA monomers which were found on the sex chromosomes of HMA-D and, at the same time, their monomer sizes fell into the required range for the analysis, i.e. below 150 pb (reflecting the size of the sequencing raw reads): HmaSat02 and HmaSat18. First, we subsampled 2 × 5,000,000 reads from each genomic library (males and females HMA-D) and aligned the reads using Bowtie2 version 1.3.1^108^ against the two aforementioned satDNA monomers. Then, we removed the central area that corresponded to one monomer and, in order to avoid sequencing error bias by CD-HIT version 4.8.1^109^, we also removed singletons (monomers detected only once) and aligned each satDNA independently using MUSCLE version 5.0^110^.

### Primer design and DNA amplification via polymerase chain reaction (PCR)

Out of the 56 HmaSatDNA monomers found by our analysis (see Supplementary Table 1), we selected 18 representative monomers (five most abundant ones and those with some differences in the abundance between sexes; indicated by asterisk in Supplementary Table 1) for physical mapping. For this purpose, we manually designed primers for each consensus monomer, using both multiple primer analyzer (Thermofisher) and OligoCalc (Biotools) to check for the hairpin formation and self-annealing. The PCR conditions followed Kretschmer et al.^67^ and it was performed in a T100 Bio-Rad Thermal Cycler (Bio-Rad Laboratories). Briefly, the amplification reaction consisted of an initial denaturation at 95 °C for 7 min, followed by 34 cycles with denaturation at 95 °C (45 s), annealing temperatures varying from 50 to 62 °C (60 s), extension at 72 °C (60 s), and the final extension at 72 °C (7 min). All PCR products were quantified using a ThermoFisher NanoDrop spectrophotometer (ThermoFisher Scientific) and submitted to electrophoresis with a 1% or 2% agarose gel (Supplementary Fig. 5).

### Fluorescence in situ hybridization (FISH)

To examine the chromosomal location of HmaSatDNA monomers, with the emphasis on their landscapes on the nascent XX/XY (HMA-C) and multiple X_1_X_1_X_2_X_2_/X_1_X_2_Y sex chromosomes (HMA-D), we performed FISH on both male and female metaphase spreads of the two karyomorphs. To unambiguously identify the sex chromosomes in both karyomorphs, we took the advantage of the previously presence of 18S ribosomal DNA (rDNA) on the neo-Y and X_1_ chromosome of HMA-D, and XY chromosomes of HMA-C^74^ and the characteristic distribution patterns of one satDNA monomer^76^. In addition, the X_2_ chromosome was identified according to its morphology and size, as it is the smallest submetacentric chromosome in the karyotype^71,74^. For FISH, we used the 18S rDNA probe (1400 pb-long segment of the 18S rRNA gene from *H. malabaricus*) was previously isolated by Cioffi et al.^111^. Finally, we also used the sex chromosome-specific marker, HmaSat07 satDNA described in the present study, in complementary experiments. Results from the sequential experiments (i.e. on the same metaphases) with 18S rDNA or HmaSat07, and each individual satDNA are provided in Supplementary Figs. 6 and 7. The HmaSat DNAs that displayed signals on the mitotic X_1_, X_2_, or neo-Y chromosomes of HMA-D, were mapped also onto male meiotic spreads in prophase I of this karyomorph and further onto HMA-C metaphases. All the 18 tested satDNA monomers along with the 18S rDNA fragment were labeled with Atto-488-dUTP; Atto-425-dUTP (green fluorescence), or Atto-550-dUTP (red fluorescence), using a nick-translation kit (Jena Bioscience, Jena, Germany) according to the manufacturer's instructions. The hybridization procedures were conducted under high-stringency conditions. After initial slide pretreatment steps with an RNAse (10 µg/ml in 2 × SSC solution) for 1 h 30 min at 37 °C and pepsin (50 µg/mL in 10 mM HCl) for 10 min, followed by denaturation in 70% formamide at 72 °C for 3 min, 15 s, and hybridization in a 37 °C moist chamber for at least 14 h^112^. Finally, the chromosome slides were stained with 4′,6-diamidino-2-phenylindole (DAPI) solution.

### Microscopy and image processing

To verify the FISH patterns, 2n and karyotype structure of each karyomorph, we analyzed at least 30 metaphases per individual. The images were captured using an Olympus BX50 microscope (Olympus Corporation, Ishikawa, Japan) with CoolSNAP, and processed using the Image-Pro Plus 4.1 software (Media Cybernetics, Silver Spring, USA). We classified chromosomes according to their arm ratios^113^ as metacentric (m), submetacentric (sm), subtelocentric (st) or acrocentric (a).

### Supplementary Information

Supplementary Information.

## Data Availability

The datasets generated during and/or analyzed during the current study are available from the corresponding author on reasonable request. The catalog of satellite DNAs was deposited on the GenBank with Accession Numbers PP262651-PP262706, and raw reads are available in Sequence Read Archive (SRA-NCBI) under Accession Numbers SRR27786732 (male) and SRR27786731 (female). All other relevant data are within the paper and its supplementary material.
