# Supplementary material for: Satellite DNAs and the evolution of the multiple X1X2Y sex chromosomes in the wolf fish Hoplias malabaricus (Teleostei; Characiformes)
Source: Sci Rep. 2024 Sep 2;14:20402. doi: 10.1038/s41598-024-70920-7 (PMC11369246; doi:10.1038/s41598-024-70920-7)
Supplement: Supplementary file 1 — Supplementary Information. [file 41598_2024_70920_MOESM1_ESM.docx]

**Satellite DNAs and the evolution of the multiple X1X2Y sex chromosomes in the wolf fish *Hoplias malabaricus* (Teleostei; Characiformes)**

Gustavo Akira Toma, Alexandr Sember, Caio Augusto Gomes Goes, Rafael Kretschmer, Fabio Porto-Foresti, Luiz Antônio Carlos Bertollo, Thomas Liehr, Ricardo Utsunomia, Marcelo de Bello Cioffi

**Supplementary Table S1:** Main features of 56 HmaSatDNA monomers found in HMA-D. Abundance is given as the proportion of the given satDNA monomer in the analyzed libraries.

| **satDNA** | **SF** | **RUL**  **(bp)** | **Abundance Female** | **Abundance Male** | **Abundance Male/Female** | **A+T**  **(%)** |
| --- | --- | --- | --- | --- | --- | --- |
| *HmaSat01 |  | 513 | 0.02135 | 0.02086 | 0.977039 | 60.4 |
| *HmaSat02 |  | 58 | 0.017459 | 0.016821 | 0.963462 | 41.4 |
| *HmaSat03 |  | 326 | 0.014004 | 0.014448 | 1.0317 | 62.9 |
| *HmaSat04 | SF1 | 280 | 0.013427 | 0.014599 | 1.087251 | 61.8 |
| *HmaSat05 |  | 613 | 0.008279 | 0.008455 | 1.021261 | 50.2 |
| HmaSat06 |  | 46 | 0.003312 | 0.003467 | 1.046931 | 47.8 |
| *HmaSat07 |  | 192 | 0.002321 | 0.002898 | 1.248987 | 64.1 |
| HmaSat08 |  | 1750 | 0.002118 | 0.002243 | 1.058812 | 61.1 |
| HmaSat09 |  | 325 | 0.001983 | 0.001734 | 0.874558 | 57.8 |
| HmaSat10 |  | 187 | 0.001981 | 0.001747 | 0.881713 | 69 |
| *HmaSat11 | SF2 | 673 | 0.001457 | 0.001048 | 0.718911 | 49.3 |
| HmaSat12 | SF3 | 30 | 0.001423 | 0.001545 | 1.085756 | 43.3 |
| HmaSat13 |  | 967 | 0.001274 | 0.001275 | 1.00064 | 64.3 |
| *HmaSat14 | SF3 | 2774 | 0.000981 | 0.001269 | 1.293461 | 65.2 |
| HmaSat15 |  | 58 | 0.000778 | 0.000854 | 1.097956 | 51.7 |
| HmaSat16 |  | 168 | 0.000682 | 0.000656 | 0.961816 | 71.4 |
| *HmaSat17 | SF1 | 152 | 0.000665 | 0.00047 | 0.706752 | 63.8 |
| *HmaSat18 | SF3 | 31 | 0.000647 | 0.001114 | 1.720524 | 48.4 |
| HmaSat19 |  | 43 | 0.000628 | 0.000645 | 1.027275 | 53.5 |
| HmaSat20 |  | 49 | 0.00059 | 0.000574 | 0.973671 | 46.9 |
| HmaSat21 |  | 322 | 0.00054 | 0.000541 | 1.003112 | 75.8 |
| HmaSat22 |  | 1040 | 0.000507 | 0.00054 | 1.064762 | 52.6 |
| HmaSat23 |  | 42 | 0.000434 | 0.000406 | 0.935339 | 52.4 |
| HmaSat24 | SF1 | 142 | 0.000431 | 0.000404 | 0.936391 | 63.4 |
| *HmaSat25 | SF2 | 972 | 0.000396 | 0.000296 | 0.747807 | 45,9 |
| HmaSat26 | SF4 | 910 | 0.000338 | 0.000338 | 1.000759 | 49.9 |
| HmaSat27 | SF5 | 237 | 0.000323 | 0.000303 | 0.937913 | 41.8 |
| HmaSat28 |  | 28 | 0.000319 | 0.000292 | 0.917733 | 67.9 |
| HmaSat29 |  | 51 | 0.000287 | 0.000321 | 1.118096 | 66.7 |
| HmaSat30 | SF4 | 403 | 0.000285 | 0.000265 | 0.930861 | 56.3 |
| HmaSat31 | SF1 | 284 | 0.000283 | 0.000309 | 1.091835 | 63.4 |
| *HmaSat32 | SF2 | 695 | 0.000276 | 0.000519 | 1.875771 | 47.9 |
| HmaSat33 | SF2 | 729 | 0.000276 | 0.000248 | 0.896593 | 50.6 |
| *HmaSat34 | SF2 | 632 | 0.000262 | 0.000336 | 1.280749 | 55.4 |
| HmaSat35 | SF2 | 1378 | 0.00025 | 0.000264 | 1.058306 | 44.1 |
| *HmaSat36 | SF2 | 1027 | 0.000226 | 0.000175 | 0.774115 | 50 |
| HmaSat37 | SF2 | 683 | 0.000217 | 0.000255 | 1.175341 | 49.5 |
| HmaSat38 |  | 27 | 0.000215 | 0.000225 | 1.047387 | 59.3 |
| HmaSat39 | SF2 | 767 | 0.000212 | 0.000219 | 1.033635 | 57.6 |
| HmaSat40 |  | 129 | 0.000195 | 0.000196 | 1.002675 | 71.3 |
| HmaSat41 |  | 176 | 0.000194 | 0.0002 | 1.029895 | 69.3 |
| *HmaSat42 | SF4 | 1756 | 0.000186 | 0.00025 | 1.340953 | 57.6 |
| HmaSat43 |  | 39 | 0.000163 | 0.000151 | 0.92382 | 38.5 |
| HmaSat44 | SF5 | 941 | 0.000159 | 0.000174 | 1.092657 | 59.4 |
| HmaSat45 |  | 199 | 0.000157 | 0.000157 | 0.997454 | 59.8 |
| HmaSat46 | SF2 | 760 | 0.000147 | 0.000131 | 0.886357 | 53.6 |
| HmaSat47 | SF2 | 1420 | 0.000145 | 0.000145 | 0.995662 | 61.4 |
| *HmaSat48 |  | 49 | 0.00014 | 0.00011 | 0.785788 | 46.9 |
| HmaSat49 |  | 174 | 0.000135 | 0.000147 | 1.090031 | 61.5 |
| HmaSat50 |  | 21 | 0.000115 | 0.000108 | 0.934986 | 71.4 |
| *HmaSat51 | SF2 | 219 | 0.000113 | 9.04E-05 | 0.800353 | 53 |
| HmaSat52 | SF4 | 211 | 0.000111 | 0.000106 | 0.951833 | 58.3 |
| HmaSat53 |  | 56 | 0.000109 | 0.000111 | 1.014982 | 53.6 |
| HmaSat54 |  | 93 | 0.000104 | 0.000102 | 0.97923 | 62.4 |
| HmaSat55 |  | 6 | 5.72E-05 | 6.10E-05 | 1.067164 | 50 |
| *HmaSat56 |  | 1760 | 4.66E-05 | 0.00015 | 3.219262 | 62.3 |

RUL: Repeat unit length; A+T: adenine and thymine content; * indicates the satDNAs chosen for the FISH mapping in this study.


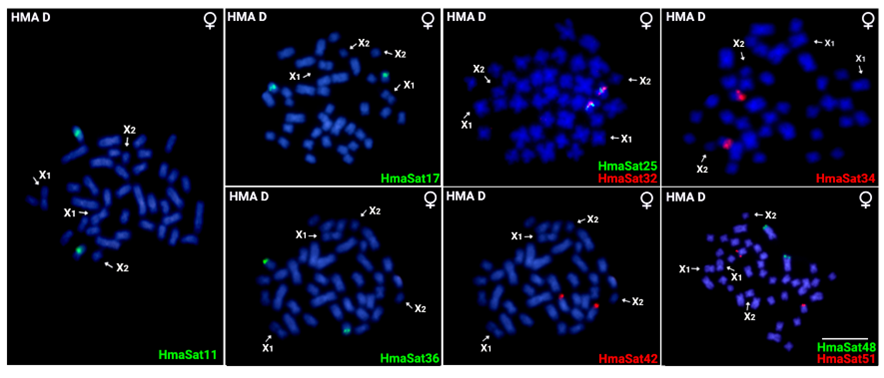


**Supplementary Figure 1:** Chromosomal location of nine HmaSatDNAs exclusively found in female autosome chromosomes of *Hoplias malabaricus* karyomorph D (2n=40). All SatDNAs family names are indicated on the right bottom, in green (Atto-488-dUTP) or red (Atto-550-dUTP). The arrows indicate the position of sex chromosomes (X_1_X_1_X_2_X_2_). Bar = 5 μm.


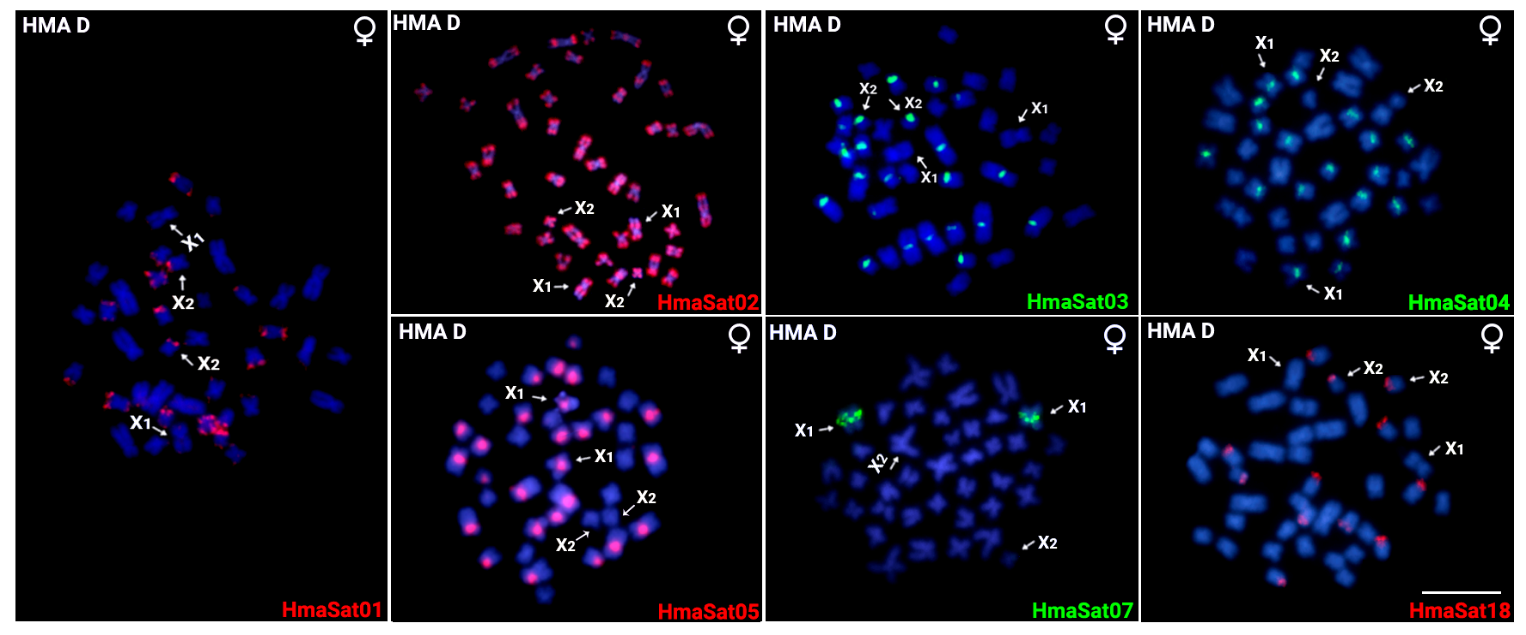


**Supplementary Figure 2:** Chromosomal location of seven HmaSatDNAs in sex chromosomes of females *Hoplias malabaricus* karyomorph D (2n=40). All SatDNAs family names are indicated on the right bottom, in green (Atto-488-dUTP) or red (Atto-550-dUTP). The arrows indicate the position of sex chromosomes (X_1_X_1_X_2_X_2_). Bar = 5 μm.


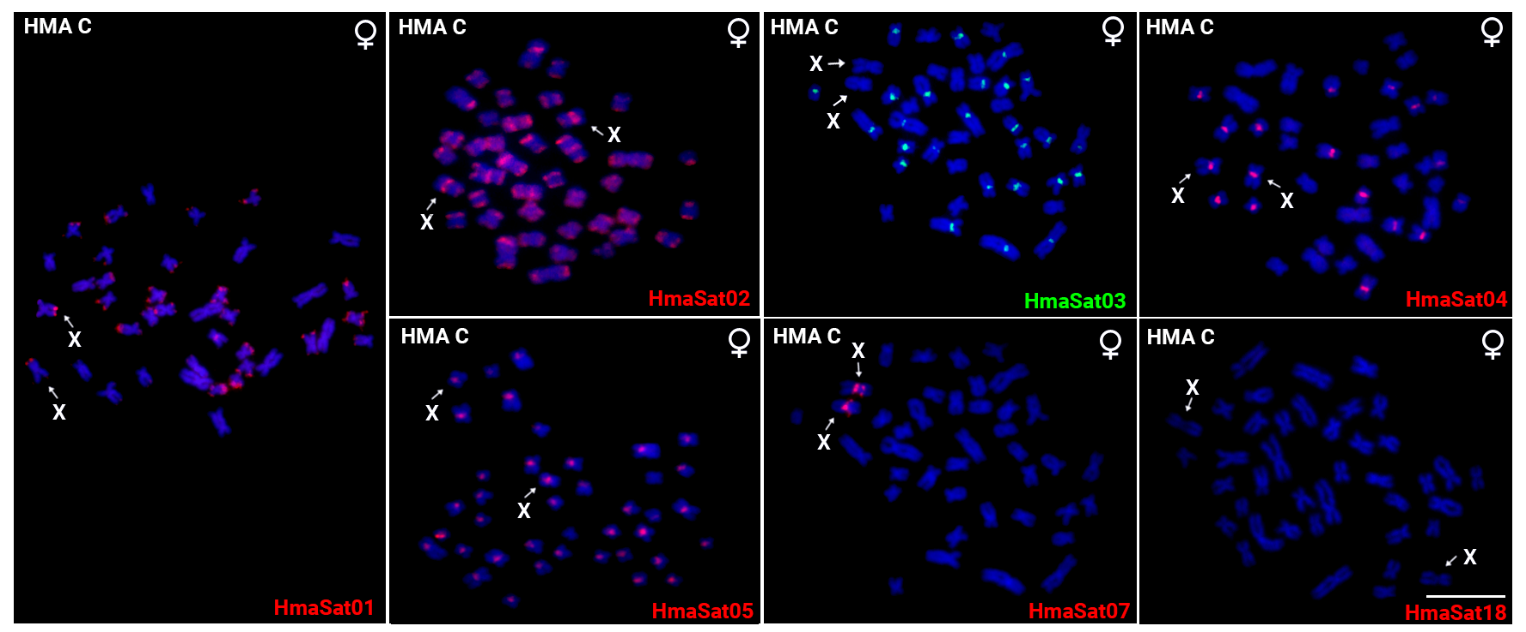


**Supplementary Figure 3:** Chromosomal location of seven HmaSatDNAs in females of *Hoplias malabaricus* karyomorph C (2n=40). All SatDNAs family names are indicated on the right bottom, in green (Atto-488-dUTP) or red (Atto-550-dUTP). The arrows indicate the position of sex chromosomes (XX). Bar = 5 μm.

**
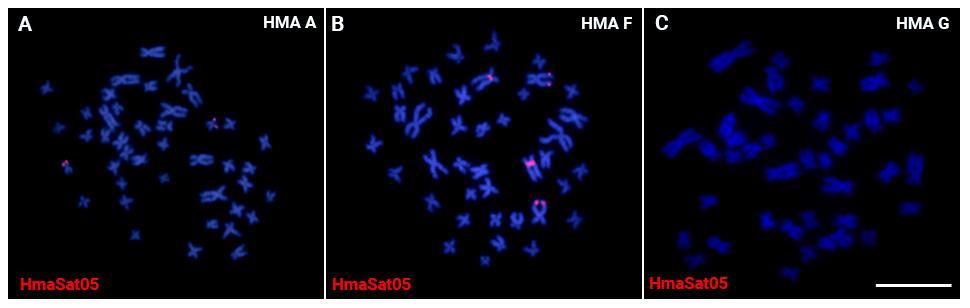
**

**Supplementary Figure 4:** Chromosomal location of the HmaSat05 in male chromosomes of *Hoplias malabaricus* karyomorph A (A), F (B), and G (C). The satDNAs family name is indicated on the left bottom, in red (Atto-550-dUTP). Bar = 5 μm.


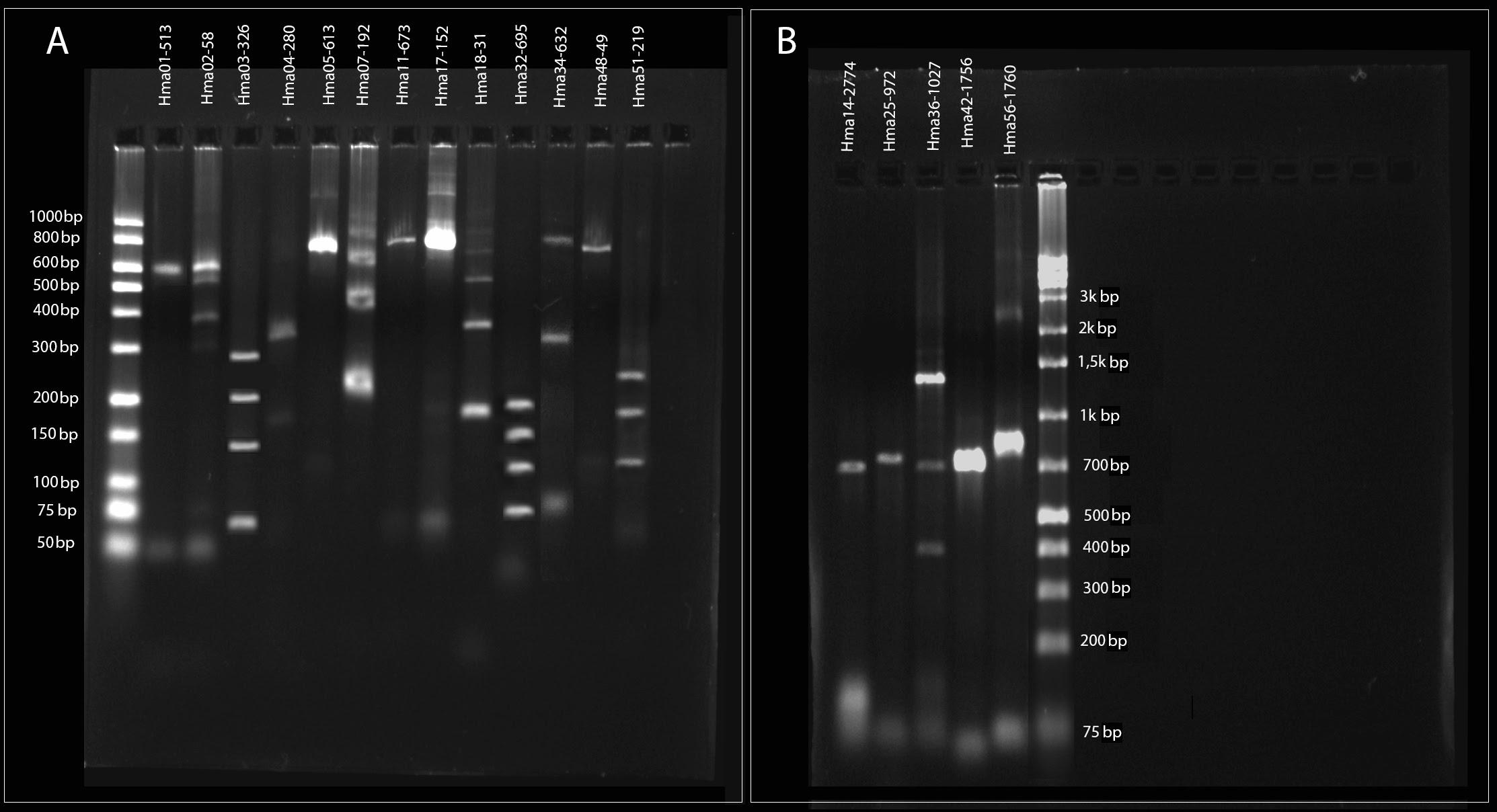


**Supplementary Figure 5:** Electrophoresis in 2% (A) and 1% (B) agarose gels, using both a 50bp low ladder (A) and 1kb ladder (B), confirming the PCRs products of HmaSatDNAs. The names of each satDNA are indicated above each well.


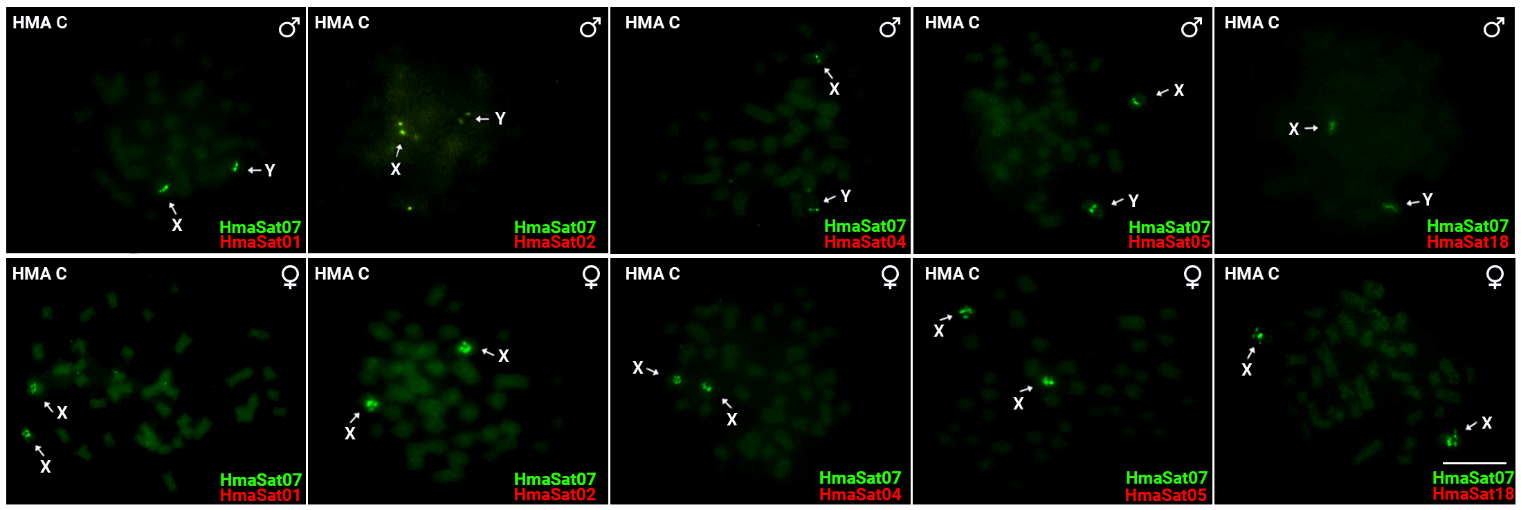


**Supplementary Figure S6**: Chromosomal location of the HmaSat07 in male and female chromosomes of *Hoplias malabaricus* karyomorph C (2n=40) used to properly identify the X and Y chromosomes. The satDNAs family names are indicated on the right bottom, in green (Atto-488-dUTP) corresponding to the HmaSat07 and in red (Atto-550-dUTP) corresponding to the previous hybridized HmaSatDNA. The arrows indicate the position of sex chromosomes. Bar = 5 μm.


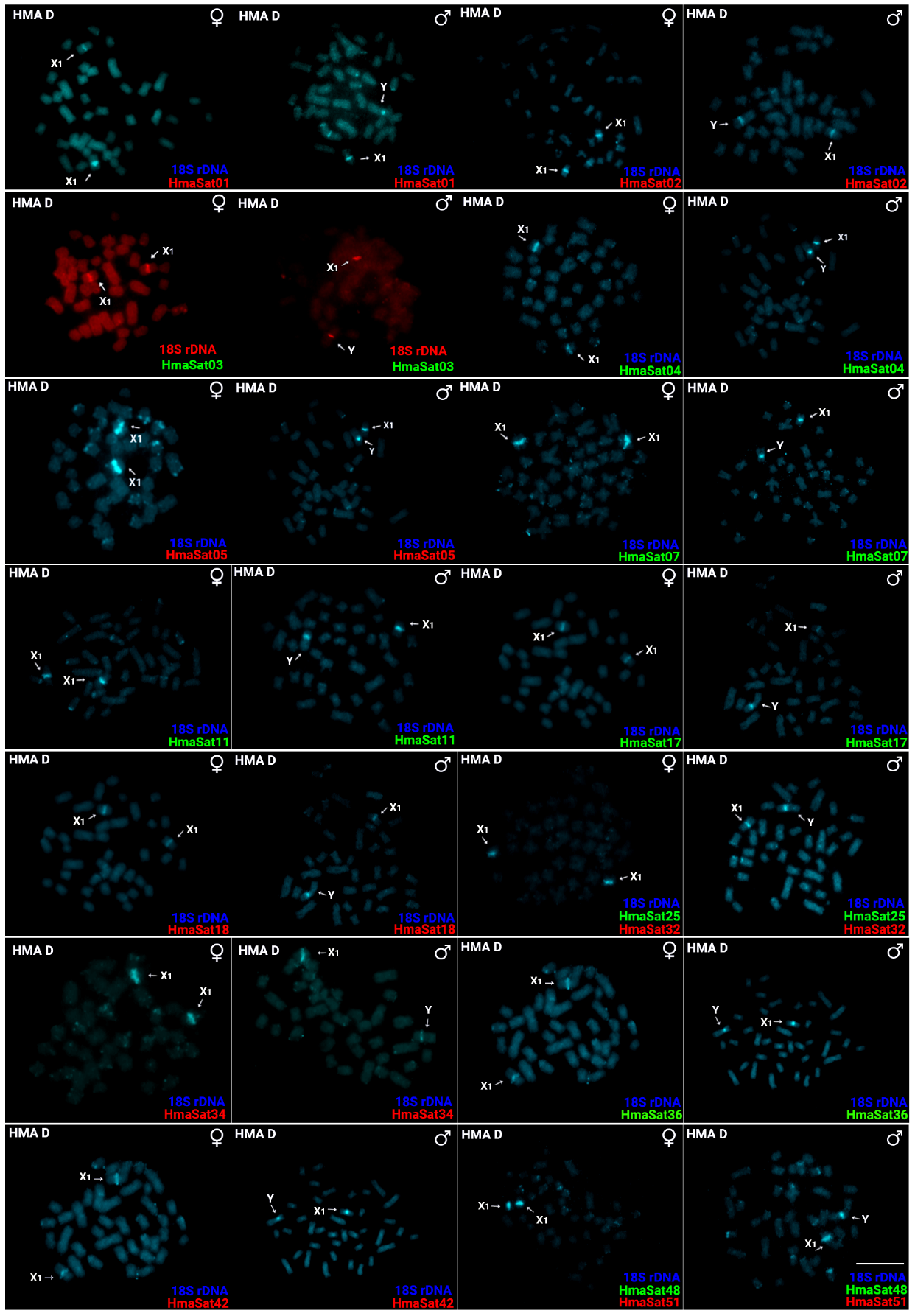


**Supplementary Figure S7:** Chromosomal location of the 18S rDNA in male and female chromosomes of *Hoplias malabaricus* karyomorph D (2n=40/39), used to properly identify the X1 and neo-Y chromosomes. The probes used are indicated on the right bottom, in blue (Atto-425-dUTP) and/or in red (Atto-550-dUTP). The arrows indicate the position of sex chromosomes (X_1_ and neo-Y). Bar = 5 μm.
